# Bovine oviductal and uterine extracellular vesicles enhance blastocyst development in confined individual embryo culture

**DOI:** 10.1007/s10815-026-03903-4

**Published:** 2026-05-16

**Authors:** Angela Travaglione, Vincenza De Gregorio, Andrea Candela, Vincenzo Genovese, Mario Cimmino, Karina Cañón-Beltrán, Yulia N. Cajas, María Iniesta-Cuerda, Ana Josefa Soler, Dimitrios Rizos, Riccardo Talevi, Roberto Gualtieri

**Affiliations:** 1 Dept Biology, University of Naples Federico II, Naples, Italy; 2 IVF Red srl, Caserta, Italy; 3 National Institute for Agriculture and Food Research and Technology (INIA-CSIC), Madrid, Spain; 4 Escuela de Ciencias Agrícolas y Ambientales, Pontificia Universidad Católica del Ecuador, Ibarra, Ecuador; 5 Department of Biological Science, Technical University of Loja, Loja, Ecuador; 6 Grupo SaBio (CSIC-UCLM-JCCM), ETSIAMB. University of Castilla-La Mancha, Albacete, Spain

**Keywords:** Extracellular vesicles, Embryo, Cattle, Individual embryo culture

## Abstract

**Purpose:**

*In vitro*-produced embryos exhibit reduced developmental competence compared to their *in vivo*-derived counterparts due to the inability to accurately replicate the complex milieu of the maternal reproductive tract. A key aspect *in vivo* is the confinement of embryos in small volumes of oviductal and uterine fluids, where maternal extracellular vesicles (EVs) deliver their cargos into developing embryos, influencing their competence. The main aim of this study was to investigate the effects of the sequential exposure to oviductal and uterine EVs on the developmental competence of bovine embryos cultured *in vitro* under different embryo densities in groups or individually.

**Methods:**

Presumptive zygotes were cultured in groups of 25 in 25 μL drops under oil (embryo density: 1/1 µL), or individually in ~ 70 nL in microwell chambers (embryo density: 14.28/1 µL), in medium alone or supplemented with oviductal EVs at days 1–4, and then with uterine EVs at days 5–8 of embryo culture.

**Results:**

Sequential EVs exposure increases the blastocyst rates, mean cell numbers, and reduces the accumulation of large detrimental lipid droplets only in individually cultured embryos. Blastocyst apoptotic cells and lipid area were reduced by sequential EVs exposure both in individually and group cultured embryos.

**Conclusions:**

We suggest that confined conditions may either enhance the accumulation of embryo-derived autocrine/paracrine factors acting synergistically with maternal EVs, or allow presumptive EV-induced embryo secretions to reach biologically effective levels only under individual culture. High-density embryo may have a key role in future culture strategies aimed to improve embryo development and competence through supplementation of bioactive maternal factors.

**Supplementary information:**

The online version contains supplementary material available at 10.1007/s10815-026-03903-4.

## Introduction

Despite advances in assisted reproductive technologies (ART), *in vitro* embryo development (IVD) remains suboptimal compared to *in vivo* conditions, resulting in reduced blastocyst quality and lower pregnancy rates [1–9]. *In vivo*, embryos develop within small volumes of oviductal fluid (OF) and subsequently uterine fluid (UF) [10]. These confined microenvironments support intense, autocrine/paracrine signaling, as well as bidirectional embryo–maternal communication, mediated by soluble factors and extracellular vesicles (EVs) containing proteins and bioactive molecules [11–14]. EVs are increasingly recognized as key mediators of intercellular communication during early reproduction and pregnancy [15–17]; these membrane-bound particles (40–1000 nm) mediate intercellular communication by carrying proteins, DNA, mRNA, lncRNA, miRNAs, and lipids [18–21], and can cross the zona pellucida to be internalized by the embryo [22, 23]. Accumulating evidence indicates that EVs participate in embryo autocrine signaling and embryo–maternal dialogue, supporting preimplantation development and developmental competence [15, 24]. Conversely, the absence of maternal-derived signals during IVD is considered a major contributor to compromised embryo quality [15]. Consistent with this view, it was recently demonstrated that EVs derived from the oviductal and uterine tracts contain distinct miRNA cargo profiles, and that sequential supplementation with oviductal- and uterine-derived EVs during group culture of bovine embryos improves blastocyst developmental competence [22, 25].

Embryo confinement and density during *in vitro* culture are also critical determinants of developmental success. IVD is consistently improved when multiple embryos are cultured together, a phenomenon known as the “group effect” observed across poly-ovulatory and mono-ovulatory species (mice [24, 26]; cattle [27, 28]; pigs [29]; cats [30]; humans [31–33]), likely due to autocrine/paracrine embryotropins that enhance embryo survival and growth [34–36]. Recent evidence shows that high-density embryo density achieved through individual culture of bovine zygotes in confined microenvironment enhances blastocyst yield and competence [37].

In this study, we demonstrate that the biological activity of maternal EVs is modulated by embryo density. Specifically, bovine zygotes were cultured to the blastocyst stage under sequential exposure to oviductal- and uterine-derived EVs. By directly comparing blastocyst yield, developmental progression, DNA fragmentation, mitochondrial activity, and lipid distribution in embryos cultured individually in an extremely small volume (1 embryo/70 nL; 14.28 embryos/µL) with those obtained from embryos cultured in groups in a larger volume (1 embryo/1 µL), we show that micro-confinement potentiates the embryonic response to sequential maternal EVs supplementation, resulting in enhanced blastocyst formation and improved developmental competence.

## Materials and methods

### EVs isolation and characterization

The isolation and characterization of EVs from maternal fluids were carried out as previously described [38]. Briefly, five oviducts and uteri were selected based on corpus luteum morphology and transported to the laboratory on ice. Oviducts (corpus luteum stage 1: days 1–4 of estrous cycle) and uterine horns (corpus luteum stage 2: day 5–10 of estrous cycle) ipsilateral to the corpus luteum were trimmed of surrounding tissues, washed with cold Ca^2+^ and Mg^2+^-free phosphate-buffered saline (PBS −) and flushed with 1 and 2.2 mL of cold PBS − respectively. After centrifugation at 300 g, 7 min, supernatants were centrifuged at 10000 g for 30 min at 4 °C, filtered through a 0.22-μm filter, and stored at 4 °C. EVs were isolated using size exclusion chromatography (SEC) (Pure EVs®, HBM-PEV; Hansa-BioMed Life Sciences, Tallinn, Estonia) followed by ultracentrifugation. Briefly, the SEC columns were washed with 30 mL PBS, loaded with each OF (≈ 1 mL) or UF (≈ 2 mL) and after adding 11 mL PBS, the first 3.0 mL of eluate was discarded and the next 2.5 mL, containing EV-rich fractions, was collected. The 2.5 mL fractions were ultracentrifuged at 100,000 g for 1 h at 4 °C (Optima L-90 K ultracentrifuge with a swinging-bucket rotor SW 41 Ti, Beckman Coulter, Fullerton, CA, USA). Pellets were resuspended in 100 μL cold PBS, pooled (final volume 500 μL) and stored at − 80 °C.

### Nanoparticle tracking analysis (NTA)

EVs concentration and size was analyzed through a NanoSight LM-10 system equipped with a CCD video camera and particle-tracking software NTA 3.1 Build 3.1.45 (NanoSight Ltd., Minton Park, UK). Five μL of EVs solution were diluted in 95μL of filtered PBS −. The NTA measurement conditions were detection threshold 2–3, camera level 13, 22 °C, 60 s. Three recordings were performed for each sample. After EVs concentration of each sample was determined, the stored pooled samples were diluted to a working concentration of 3 × 10^5^ particles/mL and used for embryo culture experiments.

### Transmission electron microscopy (TEM)

Each EVs suspension (5 μL) was diluted in 45 μL, and two 25 μL droplets of EVs preparations were adsorbed onto formvar/carbon-coated 200 mesh copper grids (Agar Scientific, Essex, UK) for 1 min at room temperature (rt). Grids were washed twice with distilled water, stained with 50μL 2% uranyl acetate for 20 s, blotted and air-dried.

### Flow cytometry

In accordance with MISEV2018 and MIFlowCyt-EV [20] guidelines, flow cytometry-based immunodetection of EV surface markers (CD9, CD63, and CD81) was performed on ten microliters from each OF-EVs and UF-EVs replicate. This previously applied approach [39] complements the structural and quantitative TEM and NTA characterization, ensuring that the isolated particles exhibit the expected EV profile in terms of size, morphology, and surface protein expression, without attempting to distinguish between EV subtypes. Analyses were performed using a CytoFLEX S flow cytometer (Beckman Coulter, Brea, CA, USA), equipped with violet (405 nm), blue (488 nm), yellow (561 nm), and red (638 nm) lasers. The cytometer was operated in low-flow mode (10μL/min), with a minimum acquisition of 10,000 events per sample. To minimize background, media were filtered through 0.1 μm membranes. To maintain optimal performance, the fluidics system was cleaned every 2–3 EVs samples with 0.1 μm-filtered distilled water, as recommended by Barranco et al. [40]. The optical configuration was optimized to detect side scatter (SSC) from the violet laser (v-SSC) and both forward scatter (FSC) and v-SSC were set to logarithmic scales. Fluorescence channels were also operated using logarithmic gains. The EV detection region in the vSSC/FSC plot was established using recombinant green fluorescent protein (GFP)-expressing exosomes (1 × 10^6^ EVs/mL; SAE0193, Merck, MA, USA). GFP fluorescence was used to define the detection threshold, and the vSSC/FSC region was adjusted to include over 90% of GFP-positive EV events. This gate was consistently applied to all EV samples, independently of fluorescence staining, ensuring that only particles within the EV size range were analyzed. CFSE staining (0.1 µM, CellTrace, Thermo Fisher Scientific, Waltham, MA, USA) was used to validate gating accuracy and evaluate sample integrity. EV samples were diluted in 0.1 µm-filtered PBS to a final concentration of 1–2 × 10⁶ EVs/mL and incubated for 30 min at 37 °C with CFSE and tetraspanin antibodies (1:50): anti-CD63-FITC (130–118-076), anti-CD81-PEVio770 (130–107–922), and anti-CD9-PEVio615 (130–118–811) from Miltenyi Biotec, Bergisch Gladbach, Germany.

Working solutions of CFSE and antibodies were centrifuged thrice (17,000 g, 10 min) to eliminate potential aggregates. After staining, 200μL of 0.1 μm-filtered PBS was added to stop the reaction. Fluorescence detection was configured as follows: CD63-FITC was excited at 488 nm and detected in the B1 channel; CD9-PEVio615 was excited at 561 nm and detected in the R2 channel (615/20 nm); CD81-PEVio770 was excited at 405 nm and detected in the B3 channel (450/50 nm). Buffer-only controls (0.1 μm-filtered PBS), secondary antibody-only controls, and unstained EV samples controls were run to verify staining specificity and rule out false-positive signals. All controls, processed under identical conditions and analyzed using the same CytoFLEX settings, confirmed the specificity of both CFSE and tetraspanin antibodies and allowed the accurate distinction between true EV events, background noise, and potential debris. For each marker, we reported both the percentage of EVs classified as positive, defined as those events within the EV detection gate (vSSC/FSC) exhibiting fluorescence intensity above the threshold established by unstained and buffer-only controls, and the corresponding mean fluorescence intensity (MFI) within that positive population. This approach allows to identify EVs that specifically express the target marker and provides a quantitative measure of the relative marker abundance among those EVs. The percentage of positive events refers to the proportion of particles within the EV detection gate (vSSC/FSC) exhibiting fluorescence above the background threshold, which was established based on unstained and media-only controls. This allows the identification of specific marker expression and supports the reliable discrimination of EVs carrying a given surface protein from non-specific signals. The MFI provides a quantitative estimate of the relative abundance of each marker within the positive EV subpopulation, with higher MFI values indicating either greater epitope density or stronger antibody binding.

Finally, the presence of residual cellular contamination was evaluated by flow cytometry following sample permeabilization, using antibodies against BSA and calnexin as detailed in the Supplementary Methods (Section: Flow Cytometry Evaluation of Contaminants).

### Oocyte collection, *in vitro* fertilization and embryo culture

Media were purchased from Stroebech media (Copenhagen, Denmark). Cumulus-oocyte complexes (COCs) retrieved by follicles (2–8 mm in diameter) from the ovaries of mature heifers sourced from a local slaughterhouse (San Marcellino-CE, CEE accreditation number 1403/M) were matured in groups of 45–50 in 500 μL of IVM medium within Nunc wells (Nunc, Roskilde, Denmark) for 24 h at 38.5 °C, 6% CO_2_, 95% humidity.

Frozen semen from three bulls (0.5 mL straws; 20 × 10^6^ sperm per straw; post-thaw motility > 70%), from Chiacchierini (Perugia, Italy), was used for fertilization. Straws were thawed 1 min in a 37 °C water bath, and washed in 10 mL of Semen Wash medium through centrifugation at 170 g for 10 min. The pellet was resuspended in 500 µL of IVF medium, and sperm concentration was determined using a Bürker chamber. Motility was assessed through the Sperm Class Analyzer (SCA, Microptic S.L., Barcelona, Spain) in a Makler chamber at 38 °C on a Nikon TE 2000 inverted microscope (Nikon, Tokyo, Japan) equipped with a Basler A312 FC camera (Basler, Ahrensburg, Germany) and a × 10 negative phase-contrast objective. Sperm concentration was adjusted to 1 × 10^6^ motile sperm/mL. COCs and sperm were co-incubated in groups of 50 in 500 µL of IVF medium in Nunc wells for 19–22 h at 38.5 °C, 6% CO_2_, and 95% humidity.

At 18–22 h post-insemination (p.i.), presumptive zygotes were denuded by vortexing in Wash medium and cultured in groups (GC) or individually (IC) as described below. Cleavage and blastocyst rates were determined at day 3 and 8 p.i., respectively. Day 8 blastocysts were grouped as early/expanded and hatching/hatched, and processed for further analyses as described below.

### Culture devices, loading of presumptive zygotes, and embryo density

Presumptive zygotes were cultured either individually in about seventy nanoliters (IC; embryo density 14.28/µL) in microwell chambers (GERI® chambers—GENEA BIOMEDX, Sydney, Australia) or in groups of 25 in 25µL drops (GC; embryo density 1/µL) in dishes under mineral oil (Heavy Oil, Stroebech media, Copenhagen, Denmark). The GERI® chamber consists of four wells, one of which includes 16 contiguous microwells allowing spatial confinement of individual embryos. Each microwell has an inverted truncated cone shape, with a base diameter of 430 µm, an upper diameter of 500 µm, and a height of 400 µm (microwell volume = 68nL). Sixteen presumptive zygotes were loaded into microwells in a 60 µL drop of medium and then covered with 3 mL of mineral oil. The 60µL drop was removed by two repeated 60 µL aspirations with the pipette tip positioned vertically in the center of the microwell array, ensuring that each zygote is cultured individually in each ~ 70nL microwell, as previously shown through dye-loading experiments [37].

Under individual culture (IC) conditions, wells containing a blastocyst correspond to an effective density of 14.28 blastocysts/µL (1 blastocyst in 70 nL). Under group culture (GC) conditions, blastocyst density can be estimated from the blastocyst rate. For example, with a 30% blastocyst rate (7.5 blastocysts out of 25 zygotes), the resulting density is 7.5 blastocysts per 25 µL, corresponding to 0.3 blastocysts/µL. Therefore, under group culture conditions, blastocyst density (blastocyst/µL) can be calculated by dividing the observed blastocyst rate by 100.

### Experimental design

**Fig. 1:**
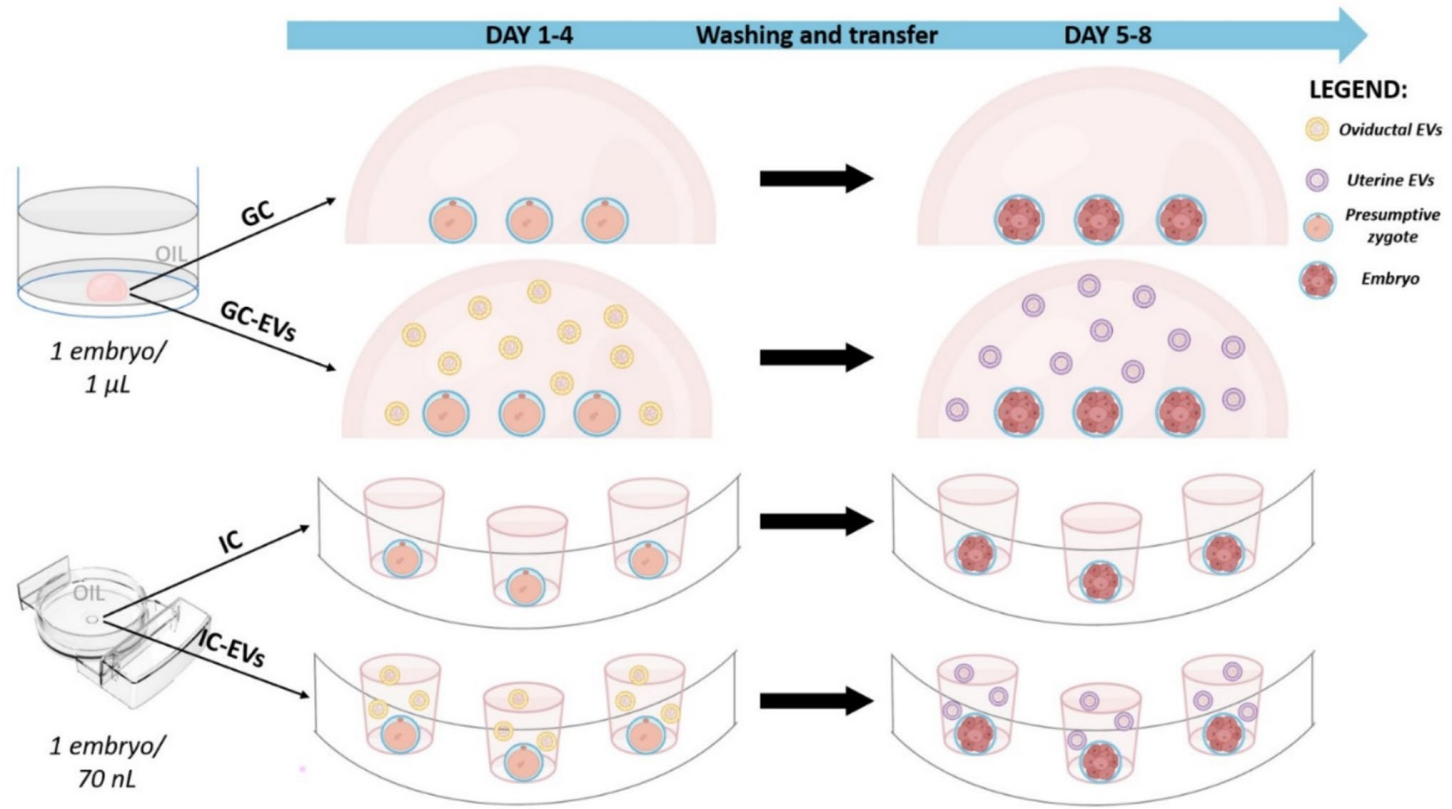
Graphic experimental design showing the embryo culture conditions and the timing of sequential exposure of individually and group-cultured embryos to oviductal and uterine EVs

Presumptive zygotes at day 1 p.i. (946 zygotes; *n* = 5 replicates) were cultured in IVC medium until day 8 p.i. in the following 4 conditions (Fig. 1): (i) GC: 25 embryos/25 µL; (ii) IC: 1 embryo/68 nL; (iii) GC-EVs: 25 embryos/25 µL supplemented with 3 × 10^5^ EVs/mL from OF (days 1–4) and 3 × 10^5^ EVs/mL from UF (days 5–8); (iv) IC-EVs: 1 embryo/68 nL supplemented with 3 × 10^5^ EVs/mL from OF (days 1–4) and 3 × 10^5^ EVs/mL from UF (days 5–8). Day 5 embryos were washed in IVC medium and transferred in new drops (GC) or microwell chambers (IC) in fresh IVC medium alone or supplemented with 3 × 10^5^ EVs/mL from UF. Day 8 blastocyst rates and stages were assessed at the stereomicroscope and blastocysts were processed as described below.

### Evaluation of blastocyst developmental competence

Developmental competence was determined through assessment of mean cell number (MCN), TUNEL-positive cells, lipid content, and mitochondrial activity. Samples were imaged at a FV3000 OLYMPUS laser-scanning confocal microscope (Olympus Italia, Segrate, Italy) and blastocyst Z-stacks were acquired using a × 30 silicone immersion objective and × 2 zoom factor.

#### Blastocyst MCN and TUNEL-positive cells

Blastocysts (GC: 34; GC-EVs: 36; IC: 31; IC-EVs: 44) fixed in 4% paraformaldehyde (PFA, Sigma Aldrich, Milan, Italy) overnight at 4 °C, were washed 3 × 5 min in phosphate-buffered saline with 0.1% polyvinylpyrrolidone (Sigma Aldrich, Milan, Italy) (PBS-PVP), permeabilized in 0.1% saponin in PBS-PVP for 30 min at rt, and incubated 1 h in the TUNEL reaction mixture (In Situ Cell Death Detection Kit, TMR red, Merck, Milan, Italy) at 37 °C in the dark, according to the manufacturer. After 3 × 5 min washes in PBS-PVP, blastocysts were stained with 10 µg/mL Hoechst 33,342 (Sigma Aldrich, Milan, Italy) in PBS-PVP for 10 min at rt, washed again, and mounted in 10µL of PBS-PVP. To preserve the three-dimensional structure of the blastocysts, two coverslips (0.17 mm thickness each) were placed on either side of the embryos to act as spacers prior to mounting. TUNEL-positive cells were visualized at 561 nm (λem 570–590 nm) and Hoechst-stained nuclei at 405 nm (λem 420–480 nm). TUNEL-positive cells and MCN were determined on maximal projection of blastocyst z-stacks through the ‘total cell counter’ tool (ImageJ 1.54f software, NIH, USA).

#### Blastocyst mitochondrial activity

Live blastocysts (GC: 15; GC-EVs: 15; IC: 17; IC-EVs: 22) were washed twice in IVC medium, incubated 30 min at 38.5 °C in 400 nM MitoTracker DeepRed (Molecular Probes, Eugene, OR, USA) in IVC medium, washed 3 × 5 min in PBS-PVP, and fixed in 4% PFA 30 min at rt. After 3 × 5 min washes in PBS-PVP, blastocysts were stained for 10 min in 10 µg/mL Hoechst 33,342 in PBS-PVP, washed 3 × 5 min in PBS-PVP, mounted on microscope slides as described above. MitoTracker DeepRed was visualized at 640 nm (λem 650–750 nm) and Hoechst-stained nuclei were visualized at 405 nm (λem 420–480 nm). Mitochondrial activity was quantified as total fluorescence intensity of maximal projections of z-stacks collected at 3 µm intervals. Blastocysts area and fluorescence integrated density (IntDen) were determined through ImageJ software. Briefly, background fluorescence of an area outside the blastocyst was subtracted, and fluorescence intensity was determined as follows: relative fluorescence = IntDen − (area of selected blastocyst × mean background fluorescence). Fluorescence intensities were expressed in arbitrary units (a.u.) [41].

#### Blastocyst lipid content

Blastocysts (GC: 16; GC-EVs: 19; IC: 19; IC-EVs: 22) fixed, washed and permeabilized as described above, were stained 1 h at rt with 20 µg/mL Bodipy (493/503, ThermoFisher Scientific, Parma, Italy) in PBS-PVP, counterstained with Hoechst 33,342, washed and mounted as described above.

Bodipy-stained lipid droplets (LDs) were visualized at 488 nm (λem 500–540 nm), and Hoechst-stained nuclei at 405 nm (λem 420–480 nm). Total lipid area was determined on three 1024 × 1024 images, one equatorial, i.e., the largest blastocyst optical section, and two in the middle of the resulting halves, for each blastocyst. Images were analyzed through the ‘nucleus counter’ tool (ImageJ 1.54f software, NIH, USA) to quantify droplet areas, and values were expressed as the area of lipid droplets relative to the total area of the blastocyst without its cavity (μm^2^). Lipid quantity was corrected by area, to account for varying blastocyst sizes [41]. For each blastocyst, after verification of a significant correlation (*r*^2^ > 0.8 and *p* < 0.0001 by Pearson’s correlation test) between lipid quantity of three sections, we chose the section with the largest area per embryo to be analyzed.

LDs number, diameter, and size frequency were evaluated on maximal projections of blastocyst z-stacks collected at 3 µm intervals. LDs parameters were measured through Image J 1.54f software (NIH, USA), as follows: background subtraction (rolling ball radius = 10), threshold adjustment (55–255), binary image conversion, watershed transformation (to remove potential LDs clusters), and ‘analyzing particles’ tool (excluding particles with the mean area < 0.07µm^2^ to avoid counting background noise). Additionally, an Excel file was created to categorize LDs size frequency into five classes: 0.07–0.3, 0.3–5, 5–15, 15–30, and > 30µm^2^.

### Statistical analysis

Each experiment consisted of at least five independent biological replicates. Blastocyst rates, developmental stages, TUNEL-positive cells, and lipid droplets (LDs) size frequencies were reported as cumulative percentages and evaluated using Fisher’s exact test for pairwise comparisons followed by Bonferroni correction for multiple testing.

Mean cell numbers (MCN), mitochondrial activity, LDs mean diameter, total number, and area were analyzed as continuous variables using one-way analysis of variance (ANOVA), followed by Tukey’s post hoc analysis, and reported as mean ± standard deviation (SD).

A *p*-value < 0.05 was considered statistically significant. Statistical analyses were performed using the GraphPad Prism software (version 8.02 for Windows, GraphPad Software, Boston, MA, USA).

## Results

### EVs characterization

Nature, size, and concentration of the EVs were analyzed through nanoparticle tracking analysis (NTA), transmission electron microscopy (TEM), and flow cytometry on pools of five samples isolated from OF and UF.

#### Nanoparticle tracking analysis

OF contained 3.32 × 10^10^ particles/mL (mean size, 237.8 nm; modal size, 174.8 nm) and UF 1.71 × 10^11^ particles/mL (mean size, 297.8 nm; modal size. 202.2 nm).

#### Transmission electron microscopy

**Fig. 2:**
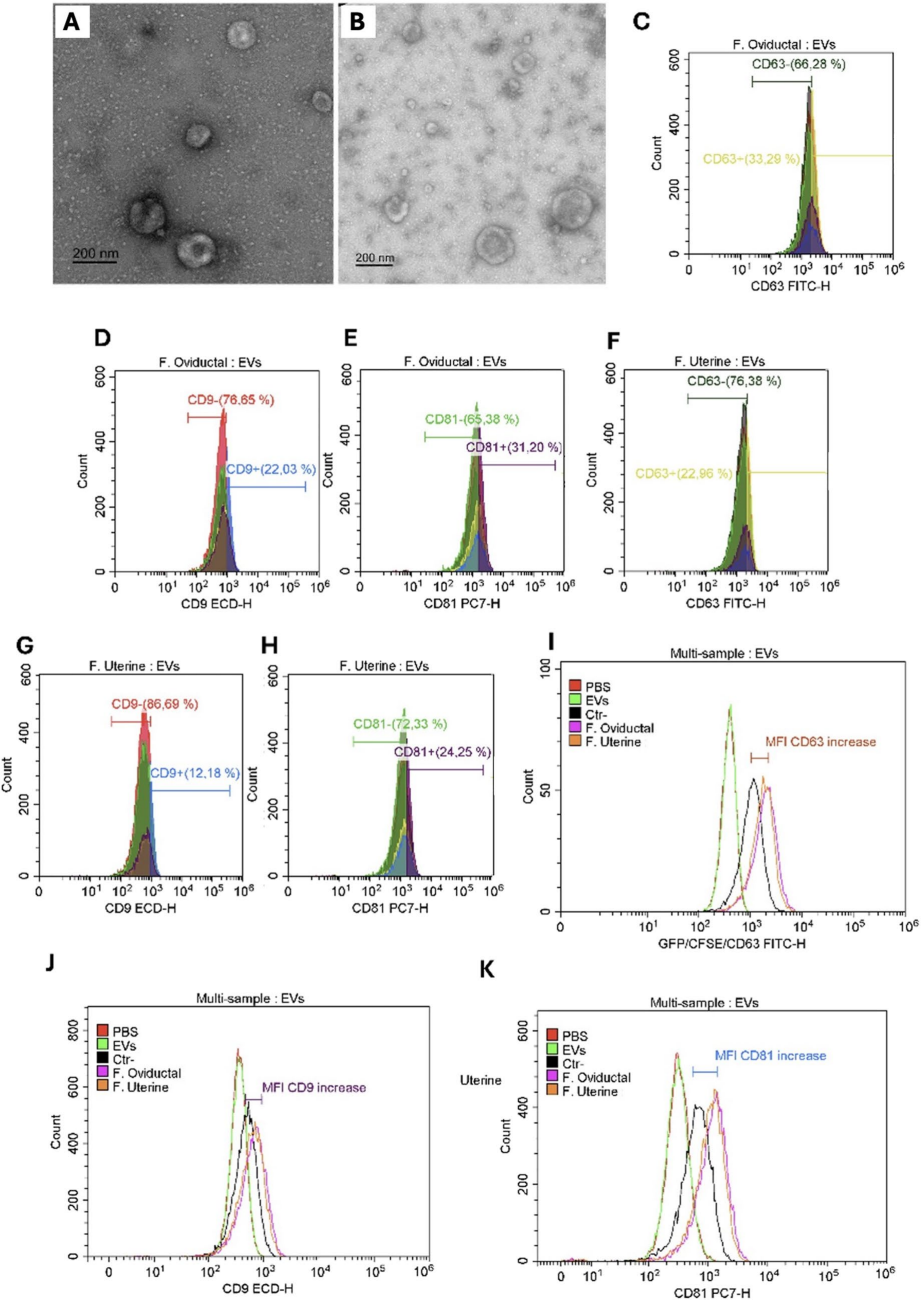
Representative TEM micrographs and flow cytometry plots of maternal extracellular vesicles (EVs) TEM revealed the typical cup-shaped morphology of EVs in oviduct (**A**) and uterus (**B**) fluids. Flow cytometry confirmed the immunopositivity of EVs for CD63, CD9, and CD81 in both oviductal (**C**–**E**) and uterine (**F**–**H**) samples. Representative histograms of fluorescence intensity for the same markers are shown in (**I** CD63; **J** CD9; **K** CD81), using the FITC, ECD, and PC7 channels, respectively. Fluorescence signals are compared to the corresponding negative controls without EVs (Ctr–, in black), PBS alone (PBS, in red), and EVs without antibodies (EVs, in green). Bar = 200 nm

TEM (Fig. 2) confirmed the characteristic cup-shaped morphology of EVs of particles in OF and UF.

#### Flow cytometry

Analyses confirmed the presence of functional and intact membrane EVs in both OF and UF. Specifically, 71.23% and 74.90% of EVs were CFSE-positive in OF and UF, respectively. The tetraspanins CD81, CD9, and CD63 were detected in OF-EVs, with EV populations (%) of CD63 (34.00 ± 3.0%), CD9 (19.39 ± 1.3%), and CD81 (31.30 ± 2.3%), and their respective signal intensities (Arbitrary Units, AU): CD63 (2977.2 ± 53.6), CD9 (1239.9 ± 101.7), and CD81 (1897.8 ± 22.3). UF-EVs displayed positive immunoreactivity for CD63 (24.70 ± 3.7%), CD9 (13.40 ± 1.7%) CD9, and CD81 (26.91 ± 3.0%), with corresponding signal intensities (AU) of CD63 (2753.9 ± 92.4), CD9 (1129.3 ± 30.6), and CD81 (1777.7 ± 52.7). These findings validated the successful isolation and integrity of EVs from both sources. Figure 2C–H shows representative flow cytometry profiles of EVs from both oviductal and uterine preparations, and panels I-K display the corresponding MFI values. The absence of cellular contaminants in the EV-enriched fractions was demonstrated by flow cytometry using antibodies against BSA and calnexin (Fig. 1 and 2 of Supplementary methods and results), confirming our previous observations [22].

### Embryo development and quality

**Fig. 3:**
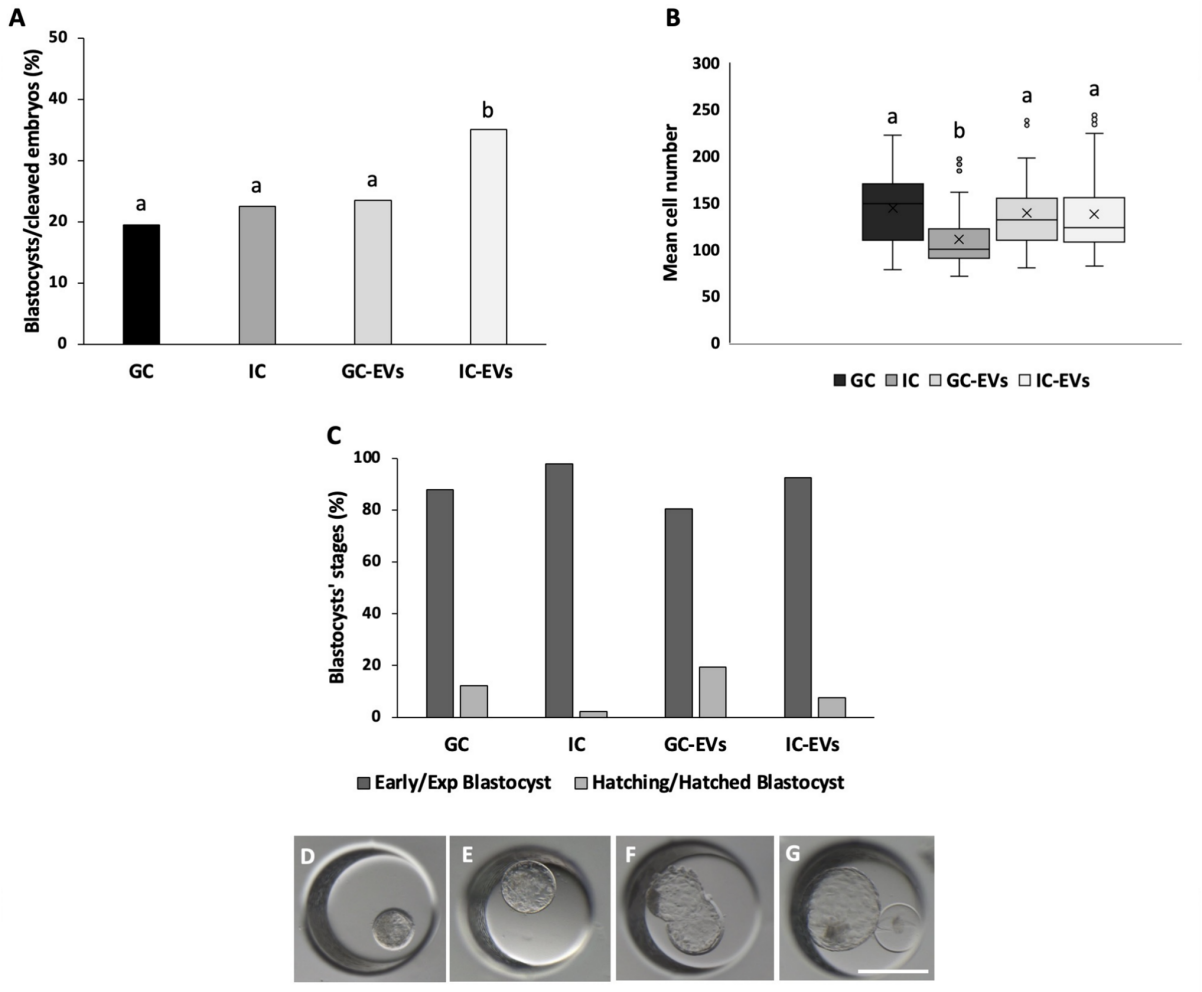
Blastocyst’s rates (**A**), MCN (**B**), and stages (**C**) at day 8 after group (GC) or individual (IC) culture in medium alone (GC, IC) or with maternal EVs (GC-EVs, IC-EVs). **D**–**G** Representative micrographs of early (**D**), expanded (**E**), hatching (**F**), and hatched blastocysts (**G**). Different superscript letters indicate significant differences: **A** *p* < 0.05. **B** GC vs IC, *p* < 0.01; IC vs GC-EVs, and IC-EVs, *p* < 0.05. C, ns. Bar = 250 mm

Cleavage rates at Day 3 p.i. ranged from 80.4% to 84.5%, showing no significant differences among the experiments. Blastocyst’s rate at day 8 p.i. (Fig. 3A) in IC-EVs (35.1%) was significantly higher compared to IC (22.5%), GC (18.9%), and GC-EVs (23.5%) (*p* < 0.001), demonstrating a positive effect of sequential EVs co-incubation on blastulation in embryos cultured individually. Although EVs increased the blastocyst rates also under group culture (blastocyst rates: GC-EVs, 23.5 versus GC, 18.9%), values were not significant. Sequential EVs co-incubation significantly increased MCN of individually cultured blastocysts (IC vs IC-EVs: 111 ± 31.7 vs 137.7 ± 46; *p* < 0.01), whereas it had no effects under the group condition (GC: 144.3 ± 42; GC-EVs: 139.2 ± 38.7) (Fig. 3B). Under our experimental conditions, the MCN of individually cultured blastocysts was significantly lower than that of group cultured blastocysts.

Assessment of blastocyst stages (Fig. 3C) was performed by distinguishing early/expanded (Fig. 3D and E) from hatching/hatched blastocysts (Fig. 3F and G). Although the percentages of hatching/hatched blastocysts were increased by sequential EVs exposure, values were not significant (Fig. 3C).

**Fig. 4:**
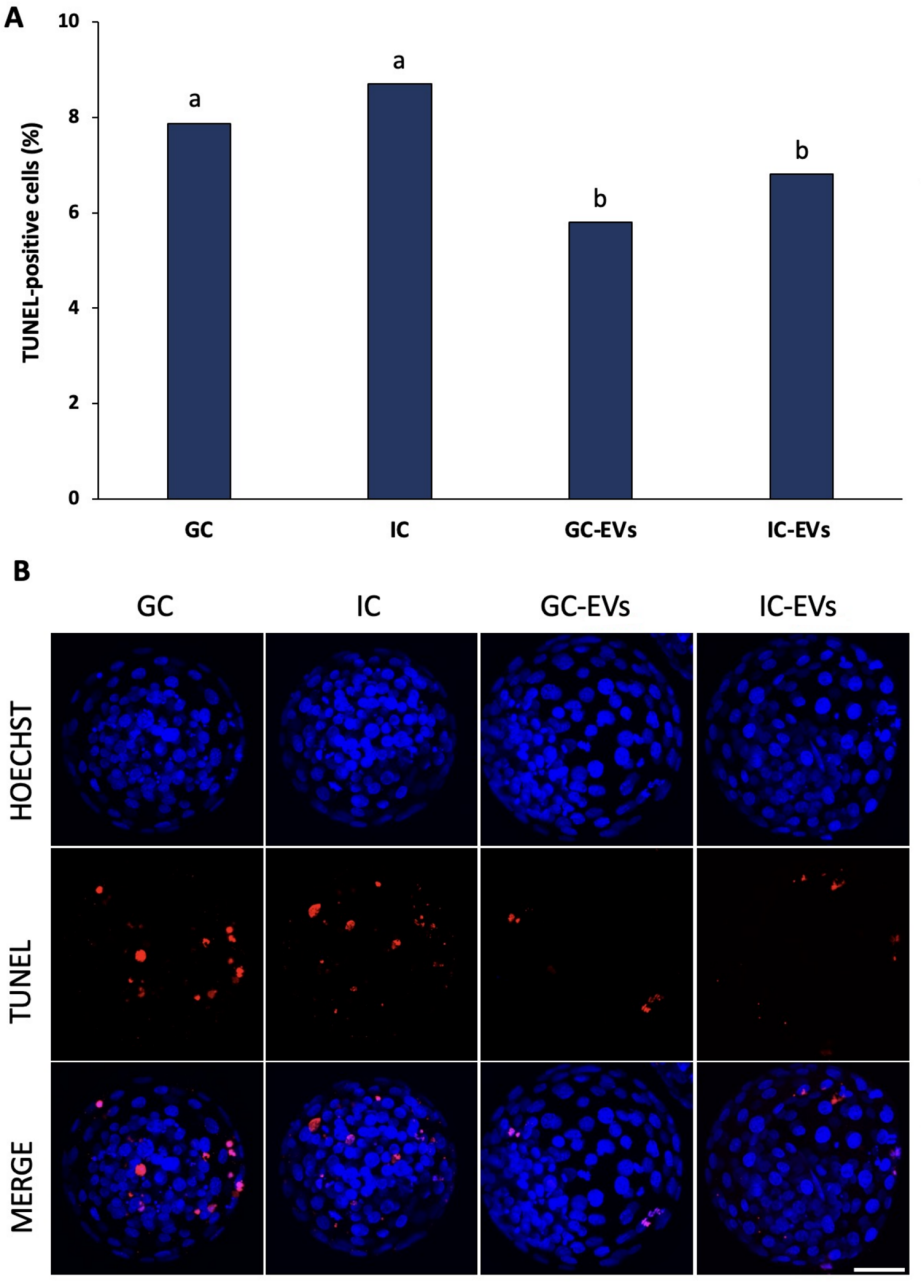
**A** TUNEL-positive cells in blastocysts. **B** Each column from left to right shows representative images of blastocysts produced in GC, IC, GC-EVs, and IC-EVs. Different superscript letters indicate significant differences: **A** GC vs GC-EVs, *p* < 0.05; IC vs IC-EVs *p* < 0.05; GC-EVs vs IC-EVs, ns. Bar = 50 μm

TUNEL-positive cells, lipid content and mitochondrial activity were assessed as markers of blastocyst developmental competence. TUNEL-positive cells in blastocysts cultured in IC-EVs (6.8%) and GC-EVs (5.8%) were significantly lower compared to IC (8.7%; *p* < 0.05) and GC (7.9%; *p* < 0.05), demonstrating a higher quality of blastocysts produced after EVs co-incubation both in individual and group culture. Although the difference was not significant, GC-EVs showed a lower percentage of apoptotic cells compared to IC-EVs (Fig. 4A). TUNEL-positive cells in blastocysts cultured individually or in groups in medium alone were not significantly different. Figure 4B shows representative images of TUNEL-positive cells (red) and total cells (blue) in blastocysts cultured under the different conditions.

**Fig. 5:**
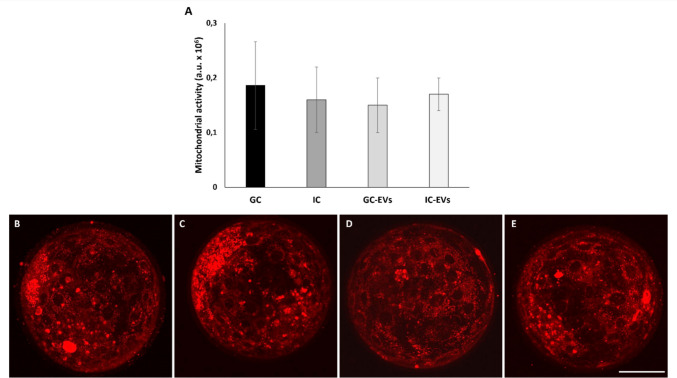
Mitochondrial activity in arbitrary units (a.u.); data are presented as mean ± SD (**A**), and representative images of blastocysts stained with MitoTracker (**B**–**E**) after group (**B**), and individual (**C**) culture in medium alone (**B**, **C**) or with maternal EVs (**D**, **E**). **A** ns. Bar = 50 µm

No significant differences in mitochondrial activity were detected in blastocysts under the different culture conditions (Fig. 5).

**Fig. 6:**
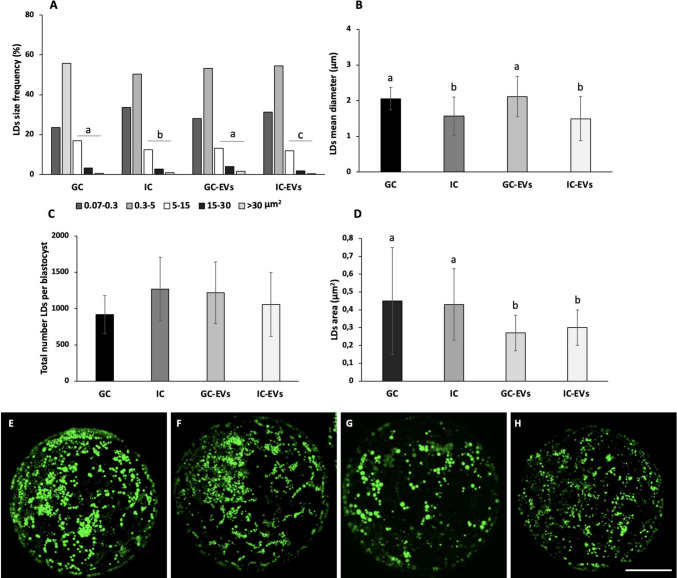
Lipid content (**A**–**D**), and representative confocal images of blastocysts produced under the different culture conditions (**E**–**H**). **A** LDs size frequency expressed in µm^2^. **B** LDs mean diameter (mean ± SD). **C** Blastocyst LDs total number (mean ± SD). **D** Blastocyst LDs area (mean ± SD). **E**–**H** Blastocysts labeled with BODIPY for LDs visualization after group (**E**,**G**) and individual (**F**, **H**) culture in medium alone (**E**, **F**) or with EVs (**G**, **H**). Different superscript letters indicate significant differences: A and B, *p* < 0.01. C, ns. D, *p* < 0.05. Bar = 50 µm

Assessment of size frequency (Fig. 6A) showed a higher number of LDs 0.07–0.3 and 0.3–5μm^2^ for all culture conditions. Blastocysts individually cultured with EVs (IC-EVs) had a lower number of LDs from 5 to > 30μm^2^, indicative of a reduced developmental competence [7], compared to IC, GC, and GC-EVs. In agreement with previous findings (37), LDs from 5 to > 30 μm^2^ in IC were significantly lower compared to GC blastocysts. EVs exposure had no effects on LD mean diameter (Fig. 6B). However, individually cultured blastocysts had a LD mean diameter significantly lower than those cultured in groups (IC, 1.57 ± 0.5; GC, 2 ± 0.3; *p* < 0.01). Total number of LDs per blastocyst (Fig. 6C) was not significantly affected by the culture conditions. In contrast, EVs exposure significantly decreased the blastocyst area occupied by LDs (Fig. 6) both in individual and group culture.

Blastocyst rates and developmental competence are summarized in Table 1.

**Table 1:** Blastocyst rates and developmental competence

Analysis Culture condition	Blastocyst rate (%)	Mean cell number ± SD	TUNEL-positive cells (%)	Mitochondrial activity (a.u. × 106) ± SD	Lipid contentAQ			
					Large LDs frequency 5 to > 30 µm2 (%)	Mean LD diameter (µm) ± SD	Total LDs number ± SD	LDs area (µm2) ± SD
GC	18.9a	144.3 ± 42.0a	7.9a	0.186 ± 0.08a	20.7a	2.1 ± 0.3a	916.8 ± 259.9a	0.45 ± 0.3a
IC	22.5a	111.0 ± 31.7b	8.7a	0.16 ± 0.06a	16.1b	1.6 ± 0.5b	1268.7 ± 436.2a	0.43 ± 0.2a
GC-EVs	23.5a	139.2 ± 38.7a	5.8b	0.15 ± 0.05a	18.8a	2.1 ± 0.6a	1219.4 ± 425.6a	0.27 ± 0.1b
IC-EVs	35.1b	137.7 ± 46.0a	6.8b	0.17 ± 0.03a	13.0c	1.5 ± 0.6b	1053.4 ± 439.1a	0.3 ± 0.1b

Within each column, values with different superscript letters are significantly different (*p* < 0.01), except for blastocyst rate, the comparison of MCN between IC and IC-EVs, TUNEL-positive cells, and LDs area (*p* < 0.05).

## Discussion

In the present study, we investigated the effects of embryo density during sequential EVs supplementation on blastocyst yield and developmental competence. Our main finding was that EVs exposure significantly increased blastocyst rates only in individually cultured embryos at high-density, effectively rescuing zygotes that would otherwise fail to reach the blastocyst stage. This observation prompts an important question: why do EVs exert their beneficial effects exclusively under individual culture conditions, despite identical EV source, dose, and exposure schedule across culture systems? The principal factors that differentiate individual from group culture exposed to EVs are (1) a ~ 14-fold higher embryo density and a ~ 62-fold higher blastocyst density (GC-EVs blastocyst density, 0.23/mL), due to the confinement of single embryos in ~ 70 nL of culture medium, and (2) the absence of delayed or arrested companion embryos.

Two non-mutually exclusive hypotheses may account for the blastocyst rescue observed under individual culture conditions. First, the high-local concentration of embryo-derived autocrine/paracrine factors under confined individual culture may produce a combined embryotrophic effect together with maternal EVs, thereby supporting developmental progression. Embryos are known to actively shape their microenvironment by secreting factors that can be either beneficial or detrimental depending on their intrinsic quality. For example, bovine blastocysts release EVs enriched in specific miRNAs, and supplementation with selected miRNA mimics has been shown to improve embryo quality, hatching, and the expression of developmental and implantation-related genes [42, 43]. In contrast, miRNA mimics derived from delayed or arrested embryos impair development [44, 45]. Accordingly, under group culture conditions, signals released by compromised embryos may dilute or counterbalance beneficial EVs-mediated effects, preventing them from reaching the biological threshold required to rescue lower-competence embryos.

Second, maternal EVs may stimulate de novo secretion of embryo-derived autocrine factors; under individual culture, the accumulation of these EVs-induced signals could reach biologically effective concentrations, rescuing embryos that would otherwise display delayed development or arrest under group culture. Currently, direct evidence of the impact of maternal EVs on the embryonic secretome or EVs biogenesis is lacking, whereas it is becoming increasingly clear how maternal EVs influence embryo development, maternal tract function, and implantation [14, 19, 46].

In agreement with Leal et al., 2022 maternal EVs exposure in group culture produced only a modest, non-significant increase in blastocyst yield. This trend may be consistent with the same underlying mechanisms, but the substantially lower blastocyst density likely restricts the accumulation of embryo-derived autocrine/paracrine factors to effective concentrations.

Here, we assessed multiple markers of developmental competence to explore whether maternal EVs may influence embryo quality under individual and group culture conditions. Sequential exposure to EVs was associated with a higher mean cell number in blastocysts derived from individually cultured embryos, whereas no evident effect was observed under group culture conditions.

These findings appear to differ from those reported by Leal et al. (2022) [22], who observed an increase in mean cell number following sequential EV supplementation under group culture. Although our experimental approach was broadly comparable in terms of maternal fluid collection, EV preparation, and culture conditions, direct comparisons should be made with caution. At present, the reasons for this discrepancy remain unclear. Differences in embryo culture media, among other experimental variables, may have contributed to the observed discrepancies, although this was not specifically addressed in the present study.

EVs supplementation also produced a consistent anti-apoptotic effect. In both individual and group cultures, EVs exposure significantly reduced the number of TUNEL-positive cells, a recognized indicator of blastocyst competence, as developmental arrest has been associated with increased apoptosis [47]. This result agrees with previous reports showing that maternal EVs decrease blastocyst apoptosis across species and improve embryo quality [48–50].

Lipid-related parameters were also significantly affected. EVs exposure reduced the abundance of large lipid droplets in individually cultured embryos and decreased the blastocyst area occupied by lipids in both individual and group cultures. This finding is biologically relevant, as large lipid droplets (> 2 µm) are associated with reduced cryotolerance and developmental competence in bovine blastocysts [44, 51]. More broadly, these observations support previous evidence that maternal EVs modulate embryonic lipid metabolism and composition [3, 22]. In line with our results, Leal et al. [22] reported reduced lipid content in group-cultured embryos following sequential maternal EVs supplementation.

In contrast, the remaining endpoints showed only non-significant trends. EVs supplementation was associated with a higher proportion of hatching or hatched blastocysts in both individual and group culture, but these differences did not reach statistical significance. Mitochondrial activity was not significantly affected by EVs exposure under any culture condition, in agreement with previous reports [22, 37].

In conclusion, our study demonstrates that maternal EV supplementation enhances both blastocyst development and quality, with the most pronounced effects observed under confined individual culture conditions. These findings suggest that micro-confinement is not simply a physical constraint, but a functional modulator of embryo responsiveness to maternal signals, capable of rescuing embryos otherwise prone to developmental delay or arrest.

However, these results should be interpreted with caution when considering translation to human assisted reproduction. In particular, the use of high-density embryo density in extremely low culture volumes requires careful optimization, as an imbalance between embryo number and medium volume may result in nutrient depletion and the accumulation of potentially harmful metabolic by-products. Moreover, although no overt developmental impairment was observed under our experimental conditions, the possible effects of confined microenvironments on the epigenetic status of the embryo remain to be clarified.

Overall, this work provides a conceptual and experimental basis for next-generation individual embryo culture systems designed to better recapitulate the regulatory features of the maternal reproductive tract *in vitro*. Elucidating the molecular pathways underlying EVs-embryo interactions and refining confinement-based culture platforms will be key steps toward translational optimization of embryo culture strategies.

## Supplementary information

Below is the link to the electronic supplementary material.

ESM 1 (DOCX 338 KB)

## Data Availability

The data that support the findings of this study are available on request from the corresponding author, R.G.
