# Supplementary material for: Bovine oviductal and uterine extracellular vesicles enhance blastocyst development in confined individual embryo culture
Source: J Assist Reprod Genet. 2026 May 16;43(6):1921–34. doi: 10.1007/s10815-026-03903-4 (PMC13319617; doi:10.1007/s10815-026-03903-4)
Supplement: Supplementary file 1 — (DOCX 338 KB) [file 10815_2026_3903_MOESM1_ESM.docx]

**Supplementary Methods and Results: Assessment of Potential Cellular Contaminants in EV Preparations.**

To evaluate the potential presence of cellular contaminants in our EV-enriched preparations, we conducted flow cytometry analyses following sample permeabilization, using antibodies against calnexin, a well-established endoplasmic reticulum marker not expected to be present in EVs, and BSA to assess potential contamination from intracellular components. These analyses aimed to identify any residual intracellular structures or organelle fragments that may have co-purified during EV isolation. Samples were permeabilized using a mild detergent-based protocol (0.1% Triton X-100 in PBS 30 min, room temperature), which enables access to intracellular markers while preserving larger subcellular structures. This strategy has been applied in our previous flow cytometry analyses to detect intracellular markers while preserving the integrity of the cells (Peris-Frau et al., 2020; Iniesta-Cuerda et al., 2025;). Following permeabilization, samples were incubated for 1 h at room temperature with primary antibodies diluted in permeabilization buffer: anti-BSA (1:100; Sigma-Aldrich SAB1200688) and anti-calnexin (1:100; Abcam AB22595). Detection was performed using a cocktail of secondary antibodies diluted in the same buffer and incubated with the samples for an additional 30 min at room temperature (anti-mouse and anti-rabbit conjugated to Alexa Fluor 488 and Alexa Fluor 647, respectively; Abcam). To ensure specificity and validate our observations, we included the following controls: (i) permeabilization buffer alone, (ii) secondary antibodies only (without primaries), (iii) permeabilized EVs without antibodies, and (iv) unfiltered reproductive fluid without antibodies, known to contain cellular debris from the flushing procedure, that was permeabilized similarly as the EVs. Two distinct flow cytometry templates were used for analysis: A) a broad detection template with the FSC/SSC gating commonly used for detecting cells; and B) an EV-specific template, optimized for small particle detection and incorporating stringent FSC/SSC-violet (described in the Material and Methods). Thus, our approach included two levels of analysis. First, using a broader detection strategy, we compared raw reproductive fluids and EV-enriched suspensions, identifying a heterogeneous population of cells or cell-derived structures displaying distinct FSC/SSC-violet profiles, indicative of varying sizes and degrees of internal complexity (i.e., large vs. small, and complex vs. less complex populations). Under these conditions, BSA- and calnexin-positive events were readily detected in the raw reproductive fluid, averaging approximately 50% and 30%, respectively, across the four populations, whereas no detectable signal for either BSA or calnexin was observed in the EV-enriched samples (Figure 1). These results indicate that our EV preparations are free of detectable cellular contaminants, as no intracellular markers were detected under permissive conditions that successfully revealed them in unprocessed samples (raw reproductive fluid). Second, we applied the EV-specific gating template to both sample types. In this refined analysis, no BSA- or calnexin-positive events were detected in either the raw fluid or the EV-enriched samples, confirming that the events falling within the EV gate are not associated with cells or contaminating remnants (Figure 2). Together, these findings indicate the purity of the isolated vesicles and the absence of co-purified cellular contaminants.


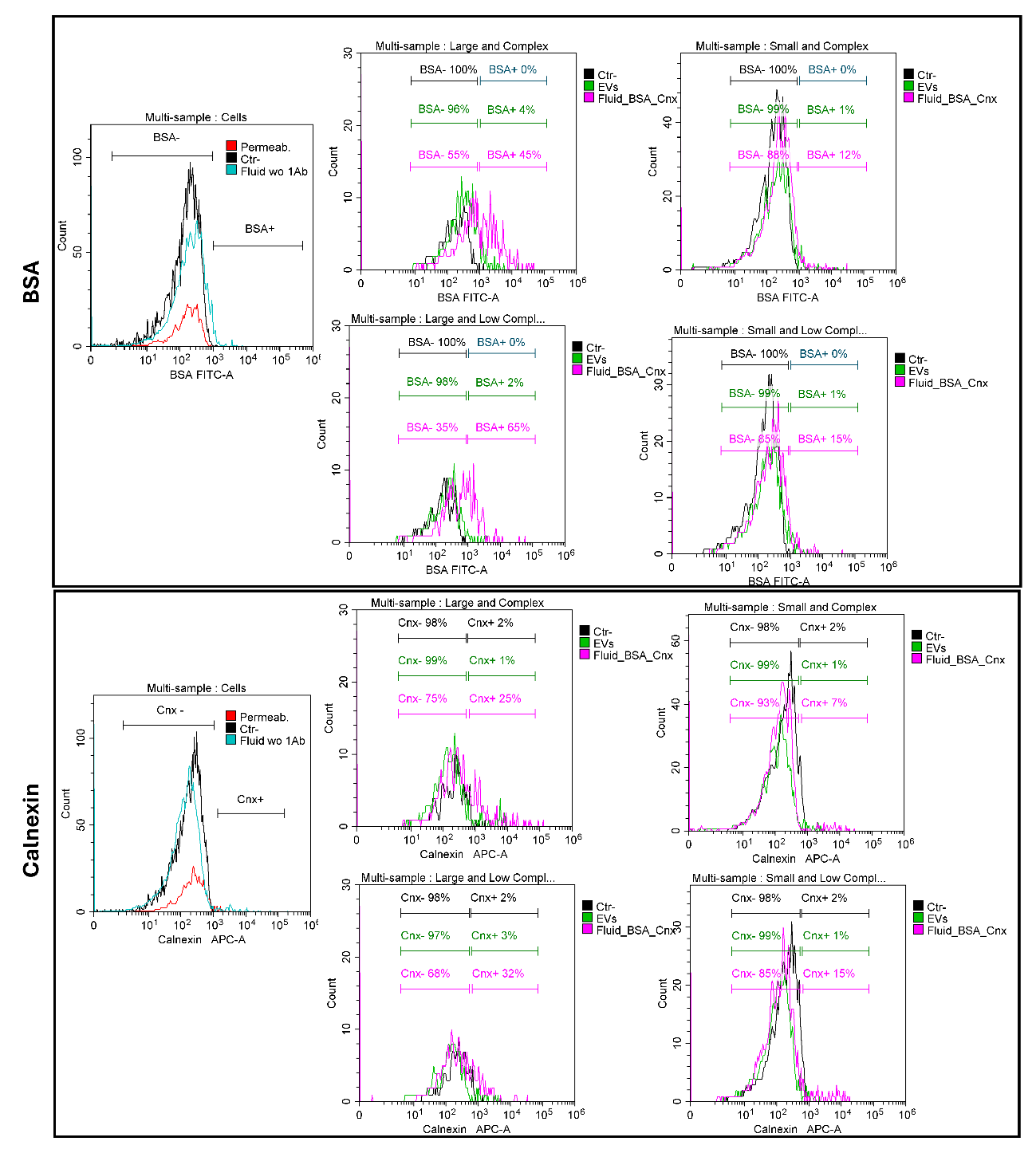


**Figure 1**. Flow cytometry analysis of BSA and calnexin in raw unfiltered reproductive fluid and purified EV samples, performed using a broad detection template with less restrictive FSC/SSC gating, typically applied for the detection of cells. Control conditions included: permeabilization buffer alone (Permab, in red); reproductive fluid incubated with secondary antibodies only, without primary antibodies, to assess nonspecific binding to fluid components (Fluid wo 1Ab, in blue); and secondary antibodies in permeabilization buffer only (Ctr–), to assess non-specific binding. BSA- and calnexin-positive events were analyzed across four particle populations defined by size (large vs. small) and internal complexity (high vs. low complexity). Populations positive for both BSA and calnexin were clearly detected in the raw reproductive fluid relative to Ctr–, while no positive events were observed in the purified EV samples, supporting the absence of contaminants of cellular origin.


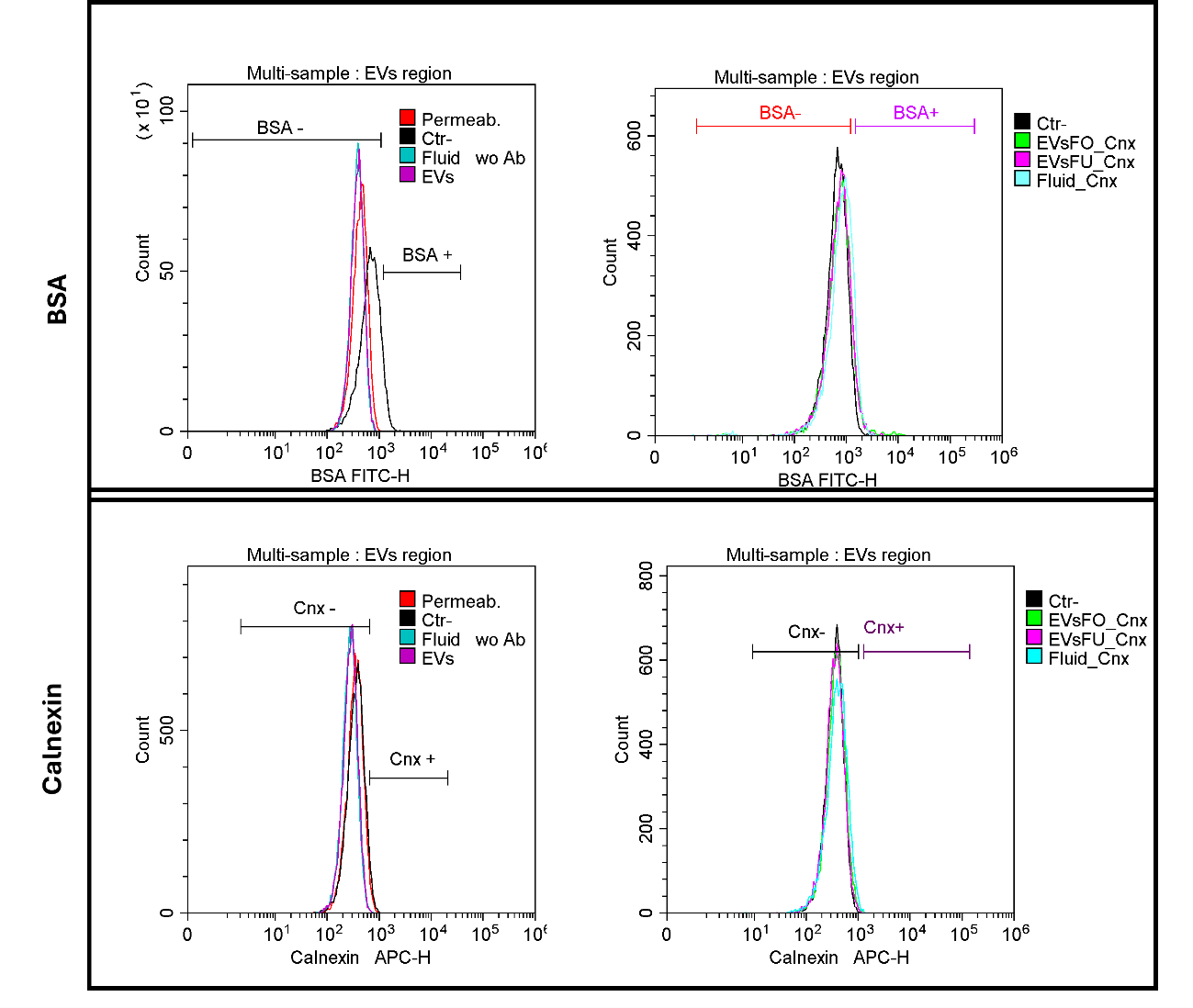


**Figure 2**. Flow cytometry analysis of BSA and calnexin in raw unfiltered reproductive fluid and purified EV samples, performed using the EV-specific detection template with stringent FSC/SSC gating optimized for small particle analysis. Control conditions included: permeabilization buffer alone (Permab, in red); secondary antibody in permeabilization buffer only (Ctr–), to assess non-specific binding; and reproductive fluid incubated with secondary antibodies only, without primary antibodies, to assess nonspecific binding to fluid components (Fluid wo 1Ab, in blue). BSA- and calnexin-positive events were analyzed within the region defined by GFP-positive events. No positive staining was detected in either the purified EV samples or the raw reproductive fluid under these conditions, indicating that particles detected within this gating region are highly likely to correspond exclusively to vesicular structures.

**References**

Iniesta-Cuerda, M., Nevoral, J., Krapf, D., Garde, J., Soler-Valls, A. J., & Yeste, M. (2025). Decoding a novel non-enzymatic protein acetylation mechanism in sperm that is essential for fertilizing potential. *Biological Research*, *58*(1). https://doi.org/10.1186/s40659-025-00613-6

Peris-Frau, P., Martín-Maestro, A., Iniesta-Cuerda, M., Sánchez-Ajofrín, I., Cesari, A., Garde, J. J., Villar, M., & Soler, A. J. (2020). Cryopreservation of ram sperm alters the dynamic changes associated with in vitro capacitation. *Theriogenology*. https://doi.org/10.1016/j.theriogenology.2020.01.046.
